# Invite everyone to the table, but not to every course: How Design-Thinking collaboration can be implemented in smart cities to design digital services

**DOI:** 10.1007/s12525-022-00567-7

**Published:** 2022-07-09

**Authors:** Frederike Marie Oschinsky, Hans Christian Klein, Bjoern Niehaves

**Affiliations:** Institute of Information Systems Research, University of Siegen, Kohlbettstrasse 15, 57072 Siegen, Germany

**Keywords:** Smart city, Smart governance, Citizen-centric government, Collaborative innovation, Design-Thinking, Digital services, O360

## Abstract

Innovative collaboration strategies are a promising tool for fostering the governance of smart cities while acknowledging citizen centricity. During implementation, however, determining the number and background of the involved actors is challenging. The Design-Thinking (DT) approach appears suitable for addressing this issue as it offers a concrete and adaptable course of action. The present contribution involves a study on implementing DT principles in a German health resort and identifies three critical components: (1) team, (2) process, and (3) workspace. Our use case is an adaptable project- and workshop plan that encourages the implementation of DT collaboration in smart cities when designing digital services. Our results provide initial guidelines on how to involve diverse actors, when to integrate trained DT coaches, and how to design collaborative innovation in a digital way. The practice-oriented insights gained in the study can be applied, adapted, and discussed in other smart cities and citizen-centered projects.

## Introduction

Today, information and communication technologies (ICT) are used in big cities and small municipalities alike for the creation of new societal developments. However, a technology-focused perspective on smart-city development often excludes citizen involvement. While an informative and constructive exchange between residents and their representatives can lead to solutions that shape life in a social, ecological, and economic sense, smart governance encourages the development of smart-living environments (D’Onofrio et al., 2019a; Pereira et al., 2018; Tomor et al., 2019). A citizen-centered approach can enable the development of new socio-economic and participatory models of society.

As tremendous need for collaborative innovation in smart cities exists (Wegrich, 2019), public managers and elected officials must make use of new problem-solving tools in order to account for today’s “wicked problems” (Linders, 2012). A multi-actor collaboration strategy in the public sector (Torfing, 2019) may help provide the information needed to develop these tools. Such a collaboration can foster “thinking outside of the box” and lead to the development of practice-oriented solutions that can be immediately tested and evaluated. The Design-Thinking (DT) approach appears suitable for implementing such a strategy and taking collaboration in smart cities to the next level because its principles (e.g., radical collaboration, experimentation, prototyping,) reflect the citizen-centered, problem-solving perspective of collaborative innovation while being concrete and adaptable.

The DT approach can be useful in addressing central challenges to today’s smart cities, including how to access, process, and use data in the urban landscape (see Finger & Portmann, 2016; Tabacchi et al., 2019). To determine the effectiveness of this approach, we investigate a joint project between seven German municipalities that utilizes in the concrete implementation of a project based on a use case. To use urban knowledge and to redesign the information exchange in these municipalities, we place equal weight on technical and human factors in the design process. Moreover, we promote transparent collaboration between partners (universities, businesses, administrations, and society).

Our analysis involves a use case in which traditional health resorts are intended to be transformed into modern health resorts as well as attractive residential and business locations. Taking good care of the citizens ‘ health and well-being, is a central task of smart cities, which is why the important area of ​​smart health has emerged. Germany has more than 350 health resorts (*Kurorte* and *Heilbäder*) that combine health services and therapies, treatment programs, naturopathic treatment, wellness programs, nutrition programs, and tourist offers. However, the number of visitors and the average length of stay has been decreasing over the past two decades, and an innovative approach to remaining an important healthcare provider is thereby needed. In redesigning these health resorts, we highlight the value of a user-centered solution that is implemented via DT collaboration. While our project clearly only represents one scenario in a small number of rural municipalities, it offers a valuable starting point for drawing conclusions about how to implement a multi-actor collaboration strategy in the public sector. Our use-case is a suitable example because the respective municipalities aim to organise, coordinate, and manage the smart redesign of their cities through joint activity.

Using information systems (IS) and Design Science Research (DSR) (Hevner et al., 2004), we derive a theory-driven, practice-oriented concept for smart cities with the aim of translating citizen centricity into action. In greater detail, we draw on the work of Peffers et al. (2007), Sonnenberg and vom Brocke (2012), and Sturm and Sunyaev (2019) in developing a DSR framework. Our research question (RQ) is:

*How can DT collaboration be implemented in smart cities in the designing of digital services?*

In answering this question, we offer three main contributions: First, we provide a toolbox for transparent and participatory collaboration in smart cities. Second, we add to literature by demonstrating how information exchange between citizens and public representatives in smart cities can be improved. Third, we contribute to existing theoretical knowledge and provide new information that can be used to inform smart-governance models. Our insights can thereby help inform future practice, design, and research.

The manuscript is structured as follows: First, we describe the current state of research and highlight the streams of collaborative innovation and DT. Second, we derive a suitable method for answering our RQ while keeping in mind our health-resort use case. Third, we switch from theory to practice and share insights on the concrete implementation of our use-case-based project. We illustrate our findings and the resulting design rationales, which lead to a guiding project plan regarding how to implement DT collaboration when designing digital services. Fourth, we discuss our insights and address exemplary solutions to the challenges as well as the limitations of our work. Finally, we provide a summary and suggest avenues for future study.

## Theoretical background

In this chapter, we briefly sum up existing literature to deduce and develop the potential of DT collaboration for a structured future-oriented progress in smart cities. To this end, we will explain the framework conditions in rural and urban areas and shed light on new forms of participation and governance. In addition, we will discuss the role of ICT in today’s smart cities and the resulting need for innovation strategies in the public sector. Moreover, we will describe the DT approach and illustrate why it offers huge potential to rethink old habits, come up with new ideas and to work with one another in an up-to-date way. It was not our aim to depict the literature in full, but rather to comprehensibly refer to the relevant aspects to answer our RQ and to illustrate our use-case example.

### Collaborative innovation in smart cities

Managing urban and rural areas is one of the most important social and economic requirements of the twenty-first century (Gil et al., 2019). This management poses challenges to infrastructure, education, health, security, and energy alike and thus goes hand in hand with vast socio-economic problems, such as resource scarcity, poverty, and the digital divide. To address these issues, local processes of societal and economic reform have been increasingly often discussed in recent years. In addition to growing research on metropolitan regions and big cities, a deeper understanding of rural regions is needed as the people who live in these regions are equally culturally diverse. Notably, however, a higher proportion of residents in rural areas are active in associations or are civically involved (Ruhlandt, 2018), which invites us to take a closer look at smart governance in these areas.

As there are comprehensive literature reviews in the field of smart city, smart city governance or participation in smart city, we will only provide a brief overview to thereupon show how our innovative approach will make the analysis, design, and development of smart cities more effective and efficient (Nilssen, 2019; Pereira et al., 2018; Shelton & Lodato, 2019; Tomor et al., 2019; Viale Pereira et al., 2017). The term “smart city” refers to developments aimed at increasing efficiency, sustainability, social inclusivity, and technological advancement in cities. Smart cities make use of ICT in order to increase the quality and efficiency of services while simultaneously reducing costs, inequality, and consumption (Yigitcanlar et al., 2018). Moreover, these cities aim to improve interactions between government, citizens, and businesses (Alawadhi et al., 2012). Due to the complex nature of smart cities, the definition of the term differs among disciplines and has evolved over time. Chourabiet al. (2012) identify eight critical factors of smart-city success (i.e., management and organization, technology, economy, infrastructure, natural environment, people and communities, policy, and governance), with smart governance representing the critical challenge that smart cities must tackle.

Governance refers to a form of governing in which a network of public- and private actors (i.e., stakeholders) share the responsibility of regulating and providing public services (Chourabi et al., 2012). The concept gained momentum in the late 1980s in response to citizens’ demand for transparency, efficiency, and legitimacy (e.g., the “Governance and Development” report by the World Bank (1992)). In the 2000s, other institutions supported strategies aimed at consolidating governance via the Web and social media (e.g., the European Union (European Governance – a White Paper, 2001)), which marked the beginning of so-called electronic governance (e-governance), or smart governance. Smart governance is defined as applying ICT in a government’s interactions with its citizens and businesses as well as in government operations (Backus, 2001). Citizen participation has become prominent (Allen et al., 2020; Sharp, 1980) in the form of input or feedback from citizens on the administration in regard to design policies, programs, and services (Feeney & Welch, 2012).

However, participation (“being involved”) has now been replaced by the demand for collaboration (“working with partners”) because public managers and elected officials need new problem-solving tools to account for today’s challenges (Linders, 2012). Although there is, to the best of our knowledge, no use case with several rural smart cities where the DT approach is used, the topic of participation is already much discussed (Gohari et al., 2020). Most of these so-called “wicked problems” cannot be appropriately tackled by traditional leadership or from a single-stakeholder perspective (Poocharoen & Ting, 2015). Instead, the concept of “vivid collaboration” was introduced and involves “(…) the process of facilitating and operating in multi-organizational arrangements to solve problems that cannot be solved or solved easily by single organizations” (Poocharoen & Ting, 2015, p. 588). The prospective aim of smart-city stakeholders is thus to constantly integrate multiple actors into their decision-making processes in order to increase value for the general public (Chatfield & Reddick, 2018; Hilgers & Ihl, 2010; Hossain & Kauranen, 2015). This citizen support can take the form of crowd sourcing, co-delivery, and reporting in addition to consultation and ideation in designing services (Allen et al., 2020). While informative and constructive exchange between residents and their representatives can lead to solutions that shape life in a social, ecological, and economic sense (D’Onofrio et al., 2019a), it is necessary to determine how smart governance can encourage the development of smart-living environments.

Throughout the evolution of smart governance, citizen centricity has remained a critical point. Although the number of smart-governance solutions and participation initiatives has increased remarkably in recent years, critics claim that technological possibilities rather than user need often determine the design of such solutions (Verdegem & Verleye, 2009). However, a technology-focused perspective of smart-city development often excludes citizen involvement, and the call for citizen-centered solutions has thus grown louder in order to increase citizens’ satisfaction and engagement (Dawes, 2008). Against this background, smart cities can be conceived as spatial units that use ICT for the progress of society and space. By using technology, governments seek to provide resources, set rules, and mediate disputes, all while empowering their citizens, unleashing social innovation, and reinvigorating democracy (see O’Reilly, 2011). The citizen-centered approach thus helps in developing new and sustainable socio-economic and participatory models of governance.

To promote innovation, society itself has become a critical source of new ideas alongside science, business, and government. Collaborative innovation represents one promising approach to strengthening citizen centricity in smart cities (Angelidou, 2015; Wegrich, 2019) and requires new infrastructures for networking, exchange, and coordination as well as new regulatory frameworks. Against this background, scientists have initiated studies on managing knowledge and innovative capabilities (Wulfsberg et al., 2016). In line with Wegrich, we define collaborative innovation as “[…] a governing arrangement where one or more public organizations engage other state or non-state stakeholders in a collective, consensus-oriented, and deliberate decision-making process with the goal to design and implement new, creative solutions to current governance challenge.” (2019, p. 12).

Collaborative-innovation strategies can help meet social needs, yet most public organizations are plagued by a scarcity of resources (Torfing, 2019). Moreover, these strategies can foster an exchange of urban knowledge and thus better tackle the aforementioned “wicked problems” because newcomers can learn from those who are more experienced at building a broad knowledge base and at allowing new ideas to emerge (ibid.). Furthermore, collaborative innovation strategies in the public sector differ from those in the private sector as they lack competition and profit motives (Roberts, 2000), which facilitates a focus on value and purpose. In addition, collective creativity (Crosby et al., 2017) is enabled by promoting perspective-taking and empathy, which allow people to share risks and fail early. The emergence of collaborative innovation has thus fundamentally changed the innovation landscape. Nevertheless, how this innovation can actually be implemented remains to be determined.

We identify and address two major challenges to collaborative innovation in smart cities. First, executives must strike a balance between homogeneity and heterogeneity in their groups (see Koppenjan & Klijn, 2004; Skilton & Dooley, 2010). Homogeneity results in a decreased ability to think outside the box, whereas heterogeneity may lead to chaos due to many differing viewpoints. If stakeholders’ viewpoints are too similar, fewer innovative solutions are found, but if they are too distinct, it becomes difficult to find common ground. To overcome this dilemma, we more-closely examine the different steps of the creative process: During problem solving and ideation, it is important to bring several perspectives and diverse expertise to the table (i.e., heterogeneity). During problem identification, synthesis, and implementation, it is crucial to combine ideas and develop concrete solutions that can be tested or evaluated (i.e., homogeneity). In a nutshell, after important stakeholders have been involved (i.e., they are all invited to the table), they do not all have to be present at every stage of development (i.e., they do not all have to partake in every course). Throughout the present work, we expand on this metaphor in greater detail.

Second, smart-city representatives aim to legitimize their decision-making by implementing collaborative strategies with multiple feedback loops (Allen et al., 2020). Thoughtfully improving the relationship between governments and their citizens enables governments to better justify their actions to public-sector organizations. To fulfill this social responsibility, promising scientific approaches are required that offer recommendations based on empirical evidence and that can be directly implemented. However, most of the literature either proposes conceptual work with broad claims and theoretical analysis (Wegrich, 2019), neglects close interaction with practitioners (Torfing, 2019), or offers narrow recommendations for specific techniques that do not account for the broad picture of collaboration. In order overcome these limitations, we combine theoretical and practical implications that can be applied to different target groups. The strength of our work lies in the concrete implementation of a use-case-based project in which we empower various stakeholders to design social solutions for their living environments that can be directly implemented.

### DT collaboration as multi-actor collaboration

The DT approach is suitable for implementing a multi-actor collaboration strategy in the public sector and for taking collaboration in smart cities to the next level. This addresses the need to find a suitable approach for implementing collaborative innovation approaches to involve multiple actors in decision-making processes (Torfing, 2019). DT is a practical approach that fosters innovation, and design thinkers seek to realize the citizen-centered, problem-solving perspective of collaborative innovation through concrete, agile, and adaptable working methods. The DT approach involves – inter alia – radical collaboration, human values, experimentation, drafting, prototyping, and process orientation and has tremendous potential to foster smart-city innovations. The approach comprises a concrete process for designing citizen-centered solutions. However, no common definition of DT exists in the academic literature (Liedtka, 2015). To render the concept more tangible, we briefly present the historical development of the approach.

In the twentieth century, theorists in architecture- and design schools began to examine the process of designing (Bazjanac, 1974; Liedtka et al., 2017). As linear problem-solving methods often fail when problems become complex and ambiguous, designers began to deal with increasing uncertainty and diversity, with problem-centeredness, nonlinearity, optionality, and ambiguity affecting their work (Liedtka, 2015, p. 926). As a result, Cross introduced the DT approach (Cross, 2011; Liedtka, 2015) and described how to think and work as a designer. Management science adapted the concept to business (Schön, 1983; Simon, 1967) and invited design thinkers to change the way organizations develop products, services, models, and strategies (Brown, 2008). As the transition to digital working methods resulted in an enormous need for agile management, businesses began to determine *who* should design (Owen, 2006) and to value empathy in better understanding collaborators and users (Brown, 2008; Liedtka, 2015). Not only did the private sector begin to implement DT increasingly often, but so, too, did the social and public sectors, for example, in their development of policies and services (Mintrom & Luetjens, 2016; Sirendi & Taveter, 2016). In recent years, elected officials and managers came to take on the role of agents of their citizens and opened the door to frequent innovations and new forms of governance. DT has undergone constant modification and is now used in many ways in various professions and sectors.

DT is a rich and complex process. In order to answer our RQ, we define three particularly important pillars of the approach (see Liedtka et al., 2017; Schmiedgen et al., 2016, see also Schindlholzer, 2014): 1) the team, 2) the process, and 3) the workspace. These pillars lead us to conclude that DT is successful due to its use of multidisciplinary teams, an iterative process, and an adaptable workspace. The DT approach 1) consists of teamwork. Being welcome to change and open and to experimentation is necessary. DT also requires a culture that views mistakes as learning opportunities (“fail early and often” (see for a further discussion Schön, 1983)). The literature emphasizes the importance of the team (Beckman & Barry, 2007; Liedtka et al., 2017) because an interdisciplinary group can generate more as well as more-original ideas than can one single person. Bringing various perspectives to the table, sharing knowledge and expertise, and appreciating different viewpoints are tools that enable both a better understanding of the task at hand and the development of useful solutions (Liedtka et al., 2017). Engaging different stakeholders, however, goes hand in hand with a certain challenge (i.e., the balance between heterogeneity and homogeneity) that needs to be considered when addressing our RQ.

**Fig. 1:**
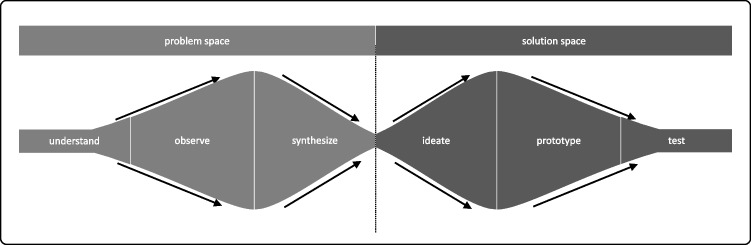
The DT steps according to Dark Horse Innovation

The DT approach 2) entails a certain process whose structure helps make people “feel comfortable in being uncomfortable” (Liedtka et al., 2017; see also Uebernickel et al., 2015) because it manages the ambiguity, complexity, and messiness of solving “wicked problems” (Liedtka et al., 2017). Although each DT school uses its own labels for the steps in the design process and subdivides them in some cases, a uniform structure of problem centricity and solution centricity can be recognized (see Fig. 1): To begin, it is important to understand the problem (understand, observe, synthesize). Next, the participants generate ideas (ideation, prototyping), which encompasses divergent and convergent thinking as well as experimenting. In the end, the participants test their most-promising ideas (testing) to evaluate their usefulness and ease of use. All steps are interconnected and can be repeated iteratively. DT provides a toolbox for every step of the process (Carlgren et al., 2016). The tools are constantly combined, expanded, and further developed in different ways and within various event formats (Elsbach & Stigliani, 2018).

In addition to accounting for the team and the process, DT highlights the importance of the workspace because the environment impacts significantly on the creative capacity of a DT group (Doorley & Witthoft, 2012). The surroundings should allow for constant interaction and collective learning and should be optimized in order provide the best environment for executing the steps in the design process. In practice, this requirement can be fulfilled by bringing easily movable furniture into largely empty rooms as well as by providing easily adaptable working material (Carlgren et al., 2016). A relaxed atmosphere (e.g., with pleasant colors, fresh air, music, and nooks in which one can retreat) is as important as supplies (e.g., healthy food and coffee, craft supplies, and protective clothing) and assistance (e.g., a help desk). Everything should be designed to be as pleasant, easeful, and uninterrupted as possible. In sum, Liedtka et al. (2017) emphasizes the notion that the collaborative DT workspace should allow for a structured DT process, a deep understanding of user context, group heterogeneity with dialogue-based conversations, and the creation of and experimentation with multiple real-world solutions.

The DT approach facilitates to come up with solution-oriented results in systems with diverse actors. For instance, business models have been developed for agile networks (Kammler et al., 2020). In addition, it has been used in public–private partnerships and increased the innovative capacity of entrepreneurship in small and medium-sized enterprises, after they were supported by scientists and public officials (Becker et al., 2020). The approach even worked well in cases where there were not many human or financial resources available (ibid.). This holds true for smart health, where cities consider the well-being of their citizens (Lepekhin et al., 2018). It worked especially well in contexts of administrative openness to change and of transparent open government partnerships (Habenstein et al., 2016). All in all, by thinking like a designer in a multi-actor environment, it became possible to develop both common values and to enable concrete practice-oriented solutions. As such, the approach has been used as an efficient method in renowned smart cities such as Bergen, Oslo, and Trondheim for a long time (Nielsen et al., 2019). All in all, we deduced our RQ from this background (i.e., “*How can DT collaboration be implemented in smart cities in the designing of digital services?”).*

## Method

Using the information-systems- (IS) and DSR paradigm (Gregor & Hevner, 2013; Hevner et al., 2004), we derive an applicable approach with which smart municipalities can translate the targeted citizen centricity into action. DSR^1^ is built on theories of design in action (Theory Type V by Gregor (2006)) that provide explicit prescriptions (e.g., methods, techniques, principles of form and function) for construction. In contrast, theories that explain, predict, or analyze – which are known from the natural and social sciences – are not yet able to develop solutions for complex situations because they do not bring something new (“artificial”) into existence, as Simon refers to it in his well-known work (Simon, 1967).

While traditional IS research focuses closely on technological artifacts, Lee et al. (2015) expanded this narrow perspective in line with the work of Hevner et al. (2004), who introduced several forms of design-science artifacts: 1) constructs, 2) models, 3) methods, and 4) instantiations. In order to provide a better understanding of artifacts, Lee et al. (2015) divide artifacts into “information artifacts” (e.g., messages), “technology artifacts” (e.g., hardware and software), and “social artifacts” (e.g., charitable acts). For our work, this approach offers a promising opportunity to understand DT collaboration in smart cities because it explicitly considers social artifacts (e.g., citizen centricity as a social artifact). In addition, the approach also maintains a technology-focused perspective on the IT artifact – which is designed via collaboration (i.e., a technology artifact) – or on its content (i.e., an information artifact). This perspective is important because the three divided artifacts can interact and result in synergies that amount to more than the sum of their parts (Lee et al., 2015).

In seeking to generate knowledge, DSR phases (e.g. Hevner et al., 2004) can be identified that are similar to those in DT process (see Footnote 1). All phases relate to iterative feedback loops to more-precisely determine either (1) what the problem is (the relevance cycle), (2) how to build and evaluate artifacts or processes (the design cycle), or (3) which experiences or expertise to consider (the rigor cycle). According to Schön (1983), who introduced the concept of reflection-in-action to the field, the timing of these loops can be varied. Building on this stance, Peffers et al. (2007) call for immediate reflection and feedback on the artifact at every stage of the design cycle. Moreover, Sonnenberg and vom Brocke (2012) introduced not only a single ex-post evaluation, but also two evaluations (ex ante and ex post) for four core design activities that are linked via evaluation (i.e., problem identification, design, construct, and use). This approach opens two doors to our work (see Hevner et al., 2004): First, we can improve rigor by adding scientific theories and methods along with domain experience into our work. Second, we can highlight relevance by demonstrating the usefulness of the artifacts’ design and by considering the requirements from the contextual environment into our research. Third, it offers pragmatic value by constantly testing and evaluating the design artifacts and processes. All in all, our project can simultaneously produce knowledge (i.e., DSR) and offer an applied procedure that enables solutions to be designed that are testable in a real-world environment (i.e., DT). Because DSR aims to explain the learnings through the process itself, we will now illustrate the concrete starting point as well as the various changes and evaluation criteria.

**Fig. 2:**
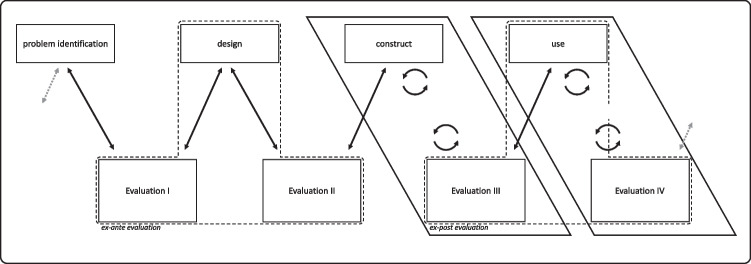
Our general DSR framework (adapted from Sonnenberg and vom Brocke (2012) )

The applied methodology consists of four design activities (c.f. Figure 2), namely: 1) problem identification, 2) design, 3) construct, and 4) use. These activities are linked via iterative evaluations. The ex-ante evaluation consists of two evaluations: Evaluation I and Evaluation II. Evaluation I informs the design activity, and Evaluation II assesses this activity. The ex-post evaluation consists of two evaluations: Evaluation III and Evaluation IV. While Evaluation III deals with the construct, Evaluation IV appraises the use of the collaborative innovation in a smart-city context and thus judges whether the solution appropriately meets the initial problem. The ex-post evaluation is conducted in a real-world setting (e.g., via workshops) and entails multiple feedback loops, which enables short evaluation cycles.

## From theory to practice: Redesigning health resorts

To challenge our theoretical assumptions, we tested them in a real-world environment. Our case of the so-called Open Government Laboratory is funded by the ministry of the interior (*Bundesministeriums des Innern und für Heimat*). Its overarching goals are to enable cooperation between administrations, businesses, and the society; to foster open governance; and to develop innovative services for smart cities. On an international level, it is a member of the international Open Government Partnership initiative. On a regional level, it embraces smart health initiatives in the rural region of Western Germany. This said, the crucial stakeholders are local administrations and politicians, clinic operators, citizens, and research institutions. Bridging the gap between public administrations and privately-run health care suppliers was done by sharing a joint vision: to design health resorts of the future, and to provide digital services. Providing these user-centric services in rural regions is complex and multi-layered – and thus, very rare to this day.

A traditional health resort from North Rhine-Westphalia in Germany approached us with severe difficulties in preparing their municipality for the future (problem identification). They were struggling to derive promising measures to react to the major trends of our time, such as sustainability and digitalization. Together, we outlined the problem and agreed on a general goal, which was to define recommendations on how traditional health resorts can be transformed into modern health resorts that can also serve as attractive residential and business locations. By using urban knowledge and redesigning the process of information exchange in these health resorts, we aimed to place equal weight on the technical and human elements of the design process. Moreover, we promoted transparent collaboration between partners (universities, businesses, administrations, and society). After a discussion, our team conducted additional literature research and discovered that there are more than 350 health resorts in Germany (*Kurorte* and *Heilbäder*) that combine health services and therapies, treatment programs, naturopathic treatment, wellness programs, nutrition programs, and tourist offers. However, the number of visitors and the average length of their stay have been decreasing over the last two decades, and an innovative approach is therefore urgently needed to come up with sustainable, economically sensible solutions. To the best of our knowledge, no best-practice example yet exists for study.

In line with the current state of research (see Chapter 2), we followed a DSR framework to refine our plan and aimed to provide a validated artifact that offers promising recommendations on how to design innovative-collaboration strategies in these areas that can be directly implemented. Again, it was critical to give equal weight to technical and human elements of the design process. For each step, we will explain why it was taken, how the criteria were selected, and how the process was defined.

After the problem had been observed and documented, we conducted additional reviews of practitioners and highlighted the need for further research. In Evaluation I, we identified collaborative innovation as an essential tool in sustainable innovations. Our evaluation criteria were applicability, suitability, novelty, economic feasibility, and importance. They were developed by Sonnenberg and vom Brocke (2012) and our literature review’s focus. This said, the criteria of applicability and importance were of particular importance to us, because they aim at receiving a justified problem statement. We therefore explicitly addressed them in our interviews in Potsdam (see below). Based on these evaluation activities, we derived initial ideas and design principles on how to implement collaborative innovation in traditional health resorts in Germany such that the people on site would feel empowered to design a health resort of the future. We discussed these propositions with various stakeholders in five different health resorts (i.e., from local administration, local companies, tourism, and gastronomy) and concluded that the DT approach could be a suitable way of tackling the identified problem. We then consulted DT experts from the Hasso-Plattner-Institute (HPI) in Potsdam, Germany, who supported our assessment. Based on this preparatory work, we sharpened our overarching RQ (i.e., “*How can DT collaboration be implemented in smart cities in the designing of digital services?”*). Moreover, we agreed on a shared first objective, which was to apply for financial support from a federal ministry. To receive this support, we submitted a project application in which we specified our core project pillars (team, process, workspace) as well as our initial project plan (design). The project application was submitted jointly by a university, six municipalities (health resorts from Stadt Bad Berleburg, Gemeinde Bad Sassendorf, Stadt Bad Laasphe, Stadt Brilon, Stadt Olsberg, and Stadt Schmallenberg) and partners from practice (Caritas Brilon für Gesundheit und Familien gGmbH, Elisabeth-Klinik gGmbH, Olsberg Ev. Johanneswerk gGmbH, Bad Berleburg Medical Park, Bad Sassendorf GmbH, VAMED Klinik, Bad Berleburg GmbH, Schmallenberger Sauerland Tourismus GmbH, BLB-Tourismus GmbH, and Winterberg Touristik und Wirtschaft GmbH). In this phase, we selected two different approaches that were proposed by Sonnenberg and vom Brocke (2012). First, we carried out a literature review to highlight the importance and relevance of our research endeavor. Second, we conducted an expert interview with a scholar from HPI about DT’s applicability in the public sector. Based on this exchange, we conducted an expert interview with an employee from a municipality to discuss our insights. Thereupon, we were able to adapt our past propositions. The most important issue was the need to be truly user- and citizen-centric.

Evaluation II was thus carried out by the ministry’s jury, which assessed the design objectives, tools, and methodology as well as the stakeholders of the design specification. The experts evaluated the various criteria (e.g., feasibility, internal consistency, clarity, completeness, and applicability) that reminded us of the work by Sonnenberg and vom Brocke (2012). Our idea was then approved for funding. However, the formal assessment was not accessible to us. After we had completed the initial phases of the DT-collaboration approach (i.e., problem identification (with Evaluation I) and design (with Evaluation II)), the project “Health Resort of the Future” (“*Kurort der Zukunft*”) was officially launched. We drafted a preliminary project plan (construct), which served as a prototype that illustrated how DT collaboration can be implemented in health resorts. We discussed this prototype in multiple feedback loops with selected stakeholders in our consortium. In this phase, we used the funder’s assessment as an evaluation. With taking their feedback very seriously, our application was successfully evaluated by the panel of experts. No changes were need, as the funding was soon approved. In the meantime, we participated in three expert workshops (Evaluation III) with renowned DT experts, namely Dark Horse Innovation in Berlin, Germany, which helped us to validate our project plan and to set it in motion (use). The proof of applicability of the prototype was based on the criteria of feasibility, ease of use, suitability, effectiveness, efficiency, compatibility with real-world phenomena, and operationality. Sonnenberg and vom Brocke’s (2012) evaluation criteria of feasibility and ease of use were of particular importance to us. We asked the experts to pay special attention to these aspects. The methods encompassed a demonstration with the prototype (i.e., project proposal) and further expert interviews in a workshop setting. In this phase, we visited several workshops by DarkHorse. The methods were diverse, but all helped identify user needs. Next, we subdivided or adapted the DT process into three steps (need findings, ideation, testing) to apply it in our use case. Sticking to this three-step process enabled us to develop own DT collaboration tools and interactive online workshops. We share our insights in the project plan.

Evaluation IV followed in a stakeholder workshop in which we implemented what we had learned about our core project pillars (team, process, workspace). The artifact paved the way for the three-year research project. The evaluation criteria were applicability, effectiveness, efficiency, compatibility, impact on the environment and user, internal consistency, and external consistency. The criteria of artifact environment and applicability were most relevant. Again, we asked the experts to pay special attention to these aspects. The validation of the artifact (i.e., the collaborative innovation as illustrated the project plan) in a naturalistic setting produced new knowledge and proved useful. In this phase, we carried out our own workshop, which we then evaluated in two ways. On the one hand, we received feedback from a quiet observation who was present throughout the whole workshop. On the other hand, we conducted semi-structured interviews after the workshop, and derived an analysis of our workshop’s strengths, weaknesses, future opportunities, and threats (i.e., SWOT). The adjustments based on this phase are found in the form of the learnings in the next section.

**Fig. 3:**
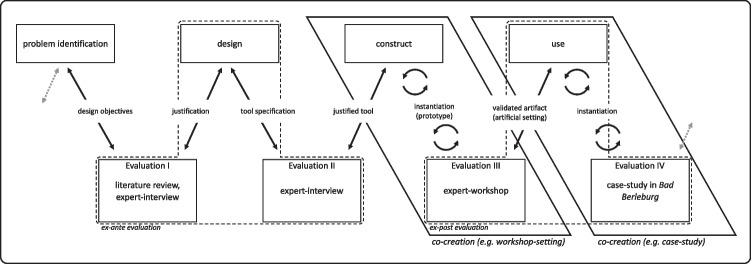
Our DSR framework for German health resorts

In our illustrative-use case, we promoted transparent collaboration between our partners and applied the DT approach to create a citizen-centered solution to redesigning these health resorts. As indicated above, we referenced Peffers et al. (2007), Sonnenberg and vom Brocke (2012) and Sturm and Sunyaev (2019) and then refined the framework (c.f. Figure 3).

The outcomes at each phase can be summarized as follows: The identification of specific challenges and needs as well as a broad literature review resulted in the overarching RQ of our work. Based on this question, we derived our research design and iteratively refined it. We summarized our approach within a project application to a federal ministry, in which we presented our overall goal and a detailed project plan. It was granted. We transferred our ideas to a real-world setting and tested the feasibility of our approach in the field. Based various feedback rounds, we successfully implemented DT collaboration in the involved municipalities.

### Implementing DT collaboration in the designing of digital services

**Fig. 4:**
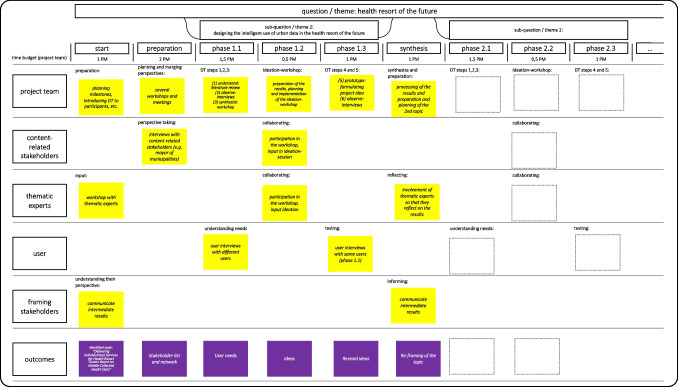
A DT-collaboration project plan for German health resorts

The use case provides an opportunity to learn and to derive recommendations for action that are useful in addressing the challenges to collaborative innovation. We again focus on the three important pillars of the DT approach (team, process, workspace) because DT is useful thanks to its use of multidisciplinary teams, an iterative process, and an adaptable workspace. We interacted closely with practitioners and citizens throughout every step in the design process to derive theoretical and practical implications as well as social solutions to citizens’ living environments that can be directly implemented. The exemplary project plan is illustrated in Fig. 4.

#### Team-oriented findings

In smart cities, multiple stakeholders are of crucial importance and need to be invited to the table. However, they do not need to participate in every step of the process, especially if they personally benefit or suffer from the solution to the problem (emotional component), if cooperating with them has been difficult in the past or could be difficult in the future (behavioral component), or if they need to be intensively trained or carefully briefed beforehand (cognitive component). DT collaboration should bear in mind that some actors have little time or prior knowledge or prefer to stick to the status quo, especially when it comes to new approaches to work (c.f. Figure 4). Political considerations play an additional role and sometimes limit the feasibility of collaboration (e.g., social desirability, proximity to elections). Consequently, public relations-, communication-, and marketing needs matter. In our project plan, we clearly defined the project team, the stakeholders, and the thematic experts. The project team’s ambidexterity comes into play in balancing administrative tasks and preparing for the changing demands of DT. To guarantee the success of collaboration, we therefore promote the inclusion of DT coaches who are open to the unexpected.

The team-oriented dimension consists of five different actors. First, the project team that consists of three research associated and one administrative employee. Together, they plan and monitor the project, and are responsible for public relation and communication measures. Second, there are content-related stakeholders such as clinic operators and smart health suppliers. The six municipalities involved act as experts for the public sector and as transfer partners who provide important feedback. Third, the users in our project are citizens, patients as well as visitors. We thereby bridge the gap between regional development, health care supply and touristic activities. Fourth, there are paramount framing stakeholders, namely the providers of our project funds. They need to be involved in a monitoring and evaluation measures. Fifth and finally, we invite further thematic experts to join the discussion. As we lacked expertise in medical informatics, the chair for microsystem design shared his expertise. In addition, we are supported by chairs for business informatics and tourism management as well as from marketing. To decide upon the question who to invite to the table, trained DT coaches can be an asset.

In each phase, the participation of different stakeholders was key (see Fig. 4). The content-related stakeholders such as clinic operators and smart health suppliers were heavily involved in Evaluation III and IV. The users mainly played a key role during problem identification and use. The framing stakeholders undertook Evaluation II and were involved in Evaluation III and IV. Further thematic experts joined the discussion when designing, constructing, and co-creating as well as in all evaluations. Finally, the project team was involved in all phases.

#### Process-oriented findings

**Fig. 5:**
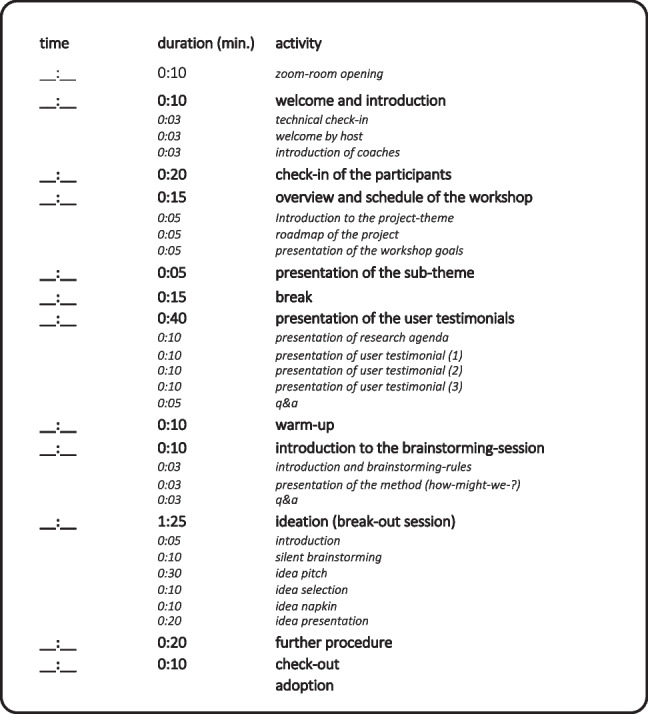
A DT-workshop approach to German health resorts

To account for the balance between heterogeneity and homogeneity, we propose including a different number of actors in the different steps of the DT process: During problem solving and ideation, it is important to bring several perspectives, user groups, and diverse expertise to the table (i.e., heterogeneity). During problem identification, synthesis, and implementation, it is crucial to combine ideas and develop concrete solutions that can be tested or evaluated (i.e., homogeneity). Again, after important actors have been involved (i.e., everyone has been invited to the table), not everyone has to be present at every stage of development (i.e., not everyone has to partake in every course). All in all, based on our insights from the project, we recommend a group size of five to six people. Additionally, we propose the use of micro-planning to comprehensibly acknowledge the different DT phases. It appears wise to involve coaches who can guarantee that the steps, tools, and feedback loops are followed and applied smoothly. Figure 5 presents such a micro-planning agenda for a workshop. Micro-planning the project plan (c.f. Figure 4) allows for a clear overview of the team-oriented time budgets (working months) and the project’s milestones.

The project plan illustrates which stakeholders need to be involved in which phase and how this can be done. In the preparation phase user reveal their needs, come up with ideas and test them. After synthesis, these steps are repeated. Whereas the project team monitors every step, the framework stakeholders are only present during kick-off and synthesis. The content-related stakeholders and thematic-experts play a crucial role in the preparation phase (e.g., in exploratory interviews and persona development with patients, tourists or inhabitants). We noticed that it makes sense to have the needs determined by a trained team, because experience and training are needed to identify specific user needs. During ideation, we then invited as many people as possible to the table. During testing, however, only users and content-related stakeholders joined. As various moderation techniques are involved, a trained team of DT coaches is a plus.

#### Workspace-oriented findings

Collaborative innovation requires a workspace. In addition to the findings from the literature, our project had to meet radically new demands because it began during a worldwide pandemic. The coronavirus (COVID-19) has clearly demonstrated that implementing digital events is a must. In DT’s newly emerging digital formats, it is important to ensure that assignments and tasks are clearly define as well as to avoid interruptions. An exemplary task is to find as many solutions as possible to a given challenge in five minutes. Because digital workshops can be quite exhaustive, the ease of use and the usefulness of the applications at hand need to be optimized, which is important in reducing participants’ digital stress or technostress and focusing on the problems and solutions at hand. This optimization includes guaranteeing that workers have enough breaks and get enough physical activity during remote work. Several experts from Dark Horse Innovation had the idea of working with Zoom (zoom.us/) and with MURAL boards (mural.co/). We also involved DT coaches with further training in digital didactics.

To illustrate how to implement DT collaboration when designing digital services, we provide a project plan below based on findings from the literature and from several iterative discussions with DT experts. This project plan illustrates our design artifact and is conceptual in nature. It is considered a DSR social artifact and brings together our considerations about people, organizations, and technology. The plan can be freely accessed upon request from the authors, reproduced, and adapted. To highlight the applicability of the project plan, we refer to a sub-question of our use case, namely a question on designing the intelligent use of urban data in a health resort of the future. This sub-question has the advantage of being neither overly broad / general nor overly narrow / specific.

## Discussion

Our results provide initial guidelines on how to involve diverse actors, when to integrate trained DT coaches, and how to design collaborative innovation in a digital way. The practice-oriented insights gained in the study can be applied, adapted, and discussed in other smart cities and citizen-centered projects. They reveal a way to effectively implement DT collaboration when designing digital services – suggesting three critical components: (1) team, (2) process, and (3) workspace.

**Fig. 6:**
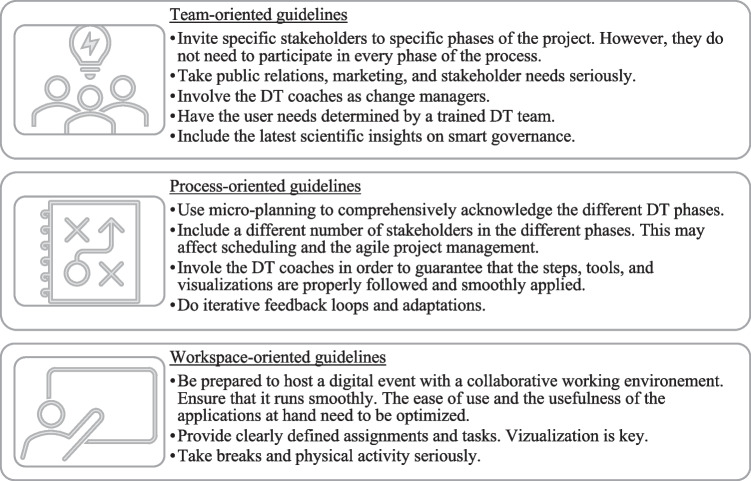
Key guidelines for implementing a DT-workshop approach

First, when dealing with smart-city developments, it is important to consider both urban and rural areas. In addition, it is necessary not only to adopt a technically driven perspective but also to include citizen centricity and the latest scientific insights in smart governance. Striking the right balance between heterogeneity and homogeneity, bridging the divide between theoretical recommendations and practical learning effects, and – finally – delivering a concept of how collaborative innovation can actually be implemented in smart cities all proved challenging. As was demonstrated, it is expedient to invite everyone to the table, but not to every course. To make collaborative innovation in smart cities more tangible, we illustrated the cognitive, emotional, and behavioral components of working collectively, all of which need to be considered. Our project plan serves as a social artifact that also considers the dimensions of information and technology. The three pillars of team, process, and workspace help to structure the plan. For every pillar, the implementation of iterative feedback loops and adaptations is important to account for every new challenge, including a global pandemic. The following Fig. 6 shows the most compelling guidelines that can be derived:

When addressing our RQ (i.e., “*How can DT collaboration be implemented in smart cities in the designing of digital services?*”), we noticed that participation (“being involved”) became replaced by the demand for collaboration (“working with partners”). However, collaboration at any cost neglects the fact that the constant integration of multiple stakeholders also requires enormous resources, both human and financial. This conclusion does not contradict citizen centricity; rather, it simply calls for a very precise consideration of the design of participatory models. By doing this, our work offers interesting implications for theory. The emerging domain of collaborative innovation among different stakeholders attracts significant scientific and policy attention. This paper contributes to developing a framework for DT collaboration for designing smart services and opens the door for future research on collaborative governance, knowledge sharing, citizen involvement, and transparency.

From a methodological point of view, case study analysis often faces challenges of rigor and external validity. We addressed the first criticism by following a systematic procedure. Nevertheless, generalizability is a shortcoming we are aware of and never claimed. Our study’s strength was its exploratory, insightful, and theory-building nature. As such, our structural approach and the design rationales of our artifact provided a promising starting point for future research. Bearing this in mind, we invite to test our findings in other governance cases, e.g., in complex settings, where the digital society needs to find a way to work together (e.g., district development) (D’Onofrio et al., 2019a, b; Habenstein et al., 2016). On top of that, we recommend using specific metrics for assessment and evaluation in future research. Measurable values and quantifiable outcomes will become necessary when applying our approach in different domains.

We offer relevant implications for practice, because DT collaboration can be adapted in smart cities of every scale. Of course, this involves not only in health resorts with a focus on smart health supply, but all smart city fields of action (e.g., sustainable transport, future-oriented education, commerce infrastructure). After agreeing on the “why”, the task of redesigning health resort is about the “what” and the “how”. We aimed at developing a transferable procedure. This remains challenging, as we did not focus on the development of the content of the services themselves, but rather on the collaboration and DT approach. Moreover, we focused on rural areas instead of urban areas. Because public–private partnerships that involve the society are a topic in smart cities of every size, future studies are invited to rest our approach on a larger scale. Our work – like any other – has weaknesses due to its limited scope. Conducting more workshops and iteratively revising the project plan for German health resorts can yield additional insights in the future. In addition, we are aware that every public-sector organization has individual characteristics and that our findings may not be transferable in an un-edited manner.

Our insights did not suggest that this project plan effectively addresses the demand for transparency, efficiency, legitimacy, consolidation, and consequently, comprehensive smart governance because our project represents only one scenario in a small number of rural municipalities. Nevertheless, we offer a possible point of departure and open the door to further steps toward appreciating the application of ICT in the interactions of governments with their citizens and businesses as well as in government operations. In addition, we drew conclusions about how to implement a multi-actor collaboration strategy in the public sector and how to integrate citizens into the development of governance models to better inform researchers, designers, and practitioners.

Our main contribution to theory is our use of the DSR approach, which provided an appropriate framework for conducting research on DT in the setting of public-sector organizations. Future research can build on these findings and transfer the approach to other practical applications. For practice, the most-important benefit is using DT as a collaboration strategy and bringing collaboration to public-sector organizations as well as bringing smart governance to life. DT opens the door to collaborating without previous knowledge and to adapting to tomorrow’s changing demands and questions in an agile manner. Our use-case example provided an adaptable project plan that combined our findings about the needs of teams, processes, and workspaces. Of course, our approach is only one of many possibilities. Nevertheless, we have taken a beneficial first step that can be followed by other steps in other projects. While future studies may build on or even contradict our findings, we welcome active participation in our project and new developments that change, transfer, and expand it.

## Summary

Our conceptual work based on a use case offers a first step to making smart cities more efficient, sustainable, socially inclusive, and technologically advanced. The DT approach was used in several health resorts in Germany to address various central questions, such as how to use urban knowledge and how to design information exchange between multiple stakeholders. We summarize our findings on how municipalities can make use of ICT to increase the quality and efficiency of their services, to reduce costs, and to improve interactions between government, citizens, and businesses. Our exemplary project plan can be transferred and adapted by other smart cities to guide collaborative innovation and thus serves as a transparent tool that can inspire future participatory models.

When promoting innovation, society provides essential ideas in addition to those from science, business, and the government. Citizen-centered strategies can foster an exchange of knowledge about cities and can thus better tackle “wicked problems” because these strategies allow for building a broad knowledge base and for the emergence of new ideas. Collaborative innovation thus represents a promising approach to strengthening citizen centricity; however, it requires infrastructures for networking, exchange, and coordination as well as new regulatory frameworks. Regarding the involvement of different actors, the following rule can be applied: Everyone should be invited to the table, but not everyone should partake in every course. In the future, establishing additional frameworks and guidelines as social artifacts, combining insights from different disciplines, and continuously evaluating and adapting these artifacts will lead to the further development of smart-living environments.

## Appendix

**Table:**

The following section is based on the *Theoretical Background* and structured by the order of appearance. We present the overall contribution of each publication, derive an emerged challenge, and draw conclusions for our work. Some articles can be assigned to more than one challenge. For the sake of parsimony, however, we assigned them only one.			
Subsection: Collaborative Innovation in Smart Cities			
Related literature	Overall contribution of the related literature	Emerged challenges	How this work addresses the challenges
(Gil et al., 2019)	Technology is an important aspect in smart cities and an opportunity to address today’s challenges	The concept of smart cities is broad. The focus is set on urban areas, while there are also opportunities for rural areas	Our proposal for DT collaboration is designed and tested in the context of rural areas
(Ruhlandt, 2018),	A systematic literature review that clarifies and proposes conceptual insights of smart cities		
(Nilssen, 2019)	DT can help to generate concepts, products, insights, and knowledge which is relevant for solving “wicked problems” in smart cities	Smart city governance is an important approach to address the challenges in smart cities. For smart city governance, ICTs have two implications. On the one hand, they enable participation and on the other hand, participation is required to design ICTs	We implement DT collaboration to design digital services considering ICT (e.g., new data-based services). Moreover, we use ICT (e.g., digital events) to design these services in a participatory manner
(Pereira et al., 2018)	A literature review that defines smart city governance and the role of ICT smart governance		
(Shelton & Lodato, 2019)	Discussion on the role of citizens and their participation in the context of smart cities		
(Tomor et al., 2019)	A literature review of smart governance in the context of sustainable cities. Contextual conditions are an important factor for mixed findings		
(Viale Pereira et al., 2017)	Collaboration and participation are important factors in smart cities and for smart city governance. ICT can promote collaboration		
(Yigitcanlar et al., 2018)	A literature review that clarifies the concept of smart cities and emphasizes the role of ICT		
(Alawadhi et al., 2012)	Collaboration is a main aspect to improve smart cities. It categorizes eight relevant aspects (technology, management and organization, policy context, governance, people and communities, economy, built infrastructure, natural environment)		
(Chourabi et al., 2012)	A framework for the concept of smart cities. It identifies different factors for success	Including citizens is a key challenge when designing smart cities	We included several actors in the DT process (e.g., context-related stakeholders, thematic experts, and users)
(Backus, 2001)	Definition of smart governance and the role of ICT		
(Allen et al., 2020)	Analysis of the importance of citizens’ e-participation and co-production, which is positively associated with urban services and public sector accountability		
(Sharp, 1980)	Discussion on co-production and the use of e-participation, which can lead to better services through feedback		
(Feeney & Welch, 2012)	E-participation is presented as a prominent tool. The intensity of e-participation technology use is associated with managers’ perception of outcome		
(Linders, 2012)	Examination of the role of citizens. The work proposes a unified typology for co-production in the age of social media	To achieve a sustainable success of smart city projects and to reduce the risk of failure, the co-production of many stakeholders needs to be facilitated and designed Citizen-centricity is crucial and has two ankles: We need to enable collaboration with citizens and this collaboration must address the citizens’ needs	We enabled the collaboration with citizens through our DT approach and used a user-centered perspective. We aimed for empathically addressing the users’/citizens’ needs
(Gohari et al., 2020)	Unconventional approaches outside administrative structures can be at the expense of participatory mechanisms		
(Poocharoen & Ting, 2015)	Co-productions effectiveness depends on network process, network structure, and characteristics of actors		
(Chatfield & Reddick, 2018)	Different perspectives help to create public value in disaster management		
(Hilgers & Ihl, 2010)	External collaboration and innovation help to enhance public value through citizen integration and participation		
(Hossain & Kauranen, 2015)	Crowdsourcing is a successful tool for public participation		
(D’Onofrio, Habenstein, et al., 2019; D’Onofrio, Papageorgiou, et al., 2019),	Socio-technical relation is important in smart cities. Human–machine symbiosis can lead to carefully designed smart cities		
(Verdegem & Verleye, 2009)	A technology-oriented view may lead to more efficient services; however, smart cities need more user-centricity		
(Dawes, 2008)	The use of ICT can lead to enhanced public services and improved government operations; however, technological innovation does not necessarily lead to citizen engagement		
(Angelidou, 2015)	Citizen-centricity can be addressed by collaborative innovation in smart cities. Collaboration is necessary for promising smart city solutions	Collaborative innovation is a promising approach, but its principles must be transferred to and adapted in various different contexts. It faces two main challenges: a) In multi-actor settings, it is challenging if the perspective of the actors is either too similar or too far away from each other b) Smart city representatives need appropriate approaches to justify and legitimize their decision-making processes. Collaborative innovation needs additional justification from practice	We involve different roles and stakeholders. Heterogeneous and homogeneous perspectives are generated in the different DT phases as needed In our transparent process, smart city representatives can report initial findings in an open, comprehensive way, and can thereby improve their decision-making and accountability
(Wegrich, 2019)	Collaborative innovation is not just a matter of effort and de-bureaucratization		
(Wulfsberg et al., 2016)	Managing knowledge and innovative capabilities is important in smart cities		
(Torfing, 2019)	Collaborative innovation in form of multi-actor collaboration is promising in the public sector; however, it needs additional research and “(…) in situ knowledge about what works in practice”		
(Roberts, 2000)	The public sector encounters “wicked problems” and differs from the private sector (e.g., it lacks competition and profit motives)		
(Crosby et al., 2017)	For the public sector, innovation, design, and collective creativity can help to create public value		
(Koppenjan & Klijn, 2004)	Possible tensions can arise due to different perspectives (e.g., homogeneous / heterogeneous groups)		
(Skilton & Dooley, 2010)	Homogeneous groups can lead to less creative outcomes		
Subsection: DT Collaboration as Multi-Actor Collaboration			
Related literature	Overall contribution of the related literature	Emerged challenges	How this work addresses the challenges
(Liedtka, 2015)	Clarification of the potential of DT. The work illustrates the approach’s ability to help decision-makers by reducing cognitive biases	DT is rich and complex. No common definition exists; however, we need a clarification of the concept to develop applicable solutions	Our DT collaboration approach is based on the related work and adapted to the context of DT collaboration in a smart city
(Bazjanac, 1974)	Historical precursor and clarification of the DT concept		
(Liedtka et al., 2017)	The challenges in the social sector are meaningful. DT is a suitable approach to address them		
(Cross, 2011)	Description how designers work and how creative thinking skills and processes evolve		
(Schön, 1983)	Historical precursor and clarification of the DT concept		
(Simon, 1967)	Historical precursor and clarification of the DT concept		
(Brown, 2008)	Transfer of design principles to the business world		
(Owen, 2006)	DT is a complement to science. DT is a new and creative way of decision- making and leadership, which needs a new understanding and new tools		
(Mintrom & Luetjens, 2016)	DT is an alternative for governments to collaborate with citizens in the decision-making process. It is “varied and scattered” and at risk of not being taken seriously		
(Sirendi & Taveter, 2016)	DT is a new approach for public service design. Proactive service design is proposed as a new approach		
(Schmiedgen et al., 2016)	In the private sector, DT is understood and implemented in many ways. The report clarifies that DT is used in a variety of ways	Related work on DT, which needs to be adapted to the public sector	Based on related work, our focus became set on the dimensions of team, process, and space. We identified this as a good starting point for adapting the principles of DT to design new products, services, and processes in the public sector
(Beckman & Barry, 2007)	Development of a generic innovation process of designing and learning		
(Uebernickel et al., 2015)	DT is more than a method. It is a rich approach combining innovation and new attitudes. Ambiguity and complexity are central aspects of DT		
(Carlgren et al., 2016)	A DT framework that combines different levels (i.e., themes, principles / mindests, practices, techniques)		
(Elsbach & Stigliani, 2018)	Identification how DT can produce products and services. DT tools are essential for changing culture and vice versa		
(Doorley & Witthoft, 2012)	Space is important for creative collaboration		
(Kammler et al., 2020)	Cooperation in innovation networks is promising. Design-oriented collaboration can be beneficial for such cooperation	Related work on DT reports promising use in different collaborative settings; however, it lacks smart city best practices. There is a need to study digital service design in the context of open government initiatives in rural areas	We tested DT collaboration in the context of smart cities. Moreover, we evaluated the design of digital services in the context of open government initiatives in rural areas
(Becker et al., 2020)	Open innovation is promising for small and medium-sized enterprises (SMEs). Compensation of low resources (i.e., personnel and financial capacity) is possible through DT use		
(Lepekhin et al., 2018)	Design-oriented approaches for service design are promising in smart health and other smart cities areas		
(Habenstein et al., 2016)	Development of the Open Smart City concept based on the principle of the Smart City and Open Governance / Open Government Data. Opening administrative action brings advantages		
(Nielsen et al., 2019)	DT can address emerging challenges in smart cities, and in a collaborative setting of diverse stakeholders		

## Footnotes

1 DSR and DT are often confused because they are not clearly delimited from one another. We view DSR as a scientific approach to producing knowledge – be it conceptual (e.g., theories, frameworks) or empirical (e.g., methodologies, research designs). In contrast, DT is an applied procedure that is utilized to satisfy user needs and create solutions that are testable in real-world environments.
